# Protein Carbonylation and Lipid Peroxidation in Hematological Malignancies

**DOI:** 10.3390/antiox9121212

**Published:** 2020-12-01

**Authors:** Alba Rodríguez-García, Roberto García-Vicente, María Luz Morales, Alejandra Ortiz-Ruiz, Joaquín Martínez-López, María Linares

**Affiliations:** 1 Department of Translational Hematology, Instituto de Investigación Hospital 12 de Octubre (i+12), Hematological Malignancies Clinical Research Unit H120-CNIO, CIBERONC, ES 28041 Madrid, Spain; albrod04@ucm.es (A.R.-G.); roberg09@ucm.es (R.G.-V.); marimo13@ucm.es (M.L.M.); mortiz06@ucm.es (A.O.-R.); jmarti01@med.ucm.es (J.M.-L.); 2 Department of Medicine, Medicine School, Universidad Complutense de Madrid, ES 28040 Madrid, Spain; 3 Department of Biochemistry and Molecular Biology, Pharmacy School, Universidad Complutense de Madrid, ES 28040 Madrid, Spain

**Keywords:** oxidative stress, protein carbonylation, lipid peroxidation, hematological malignancies

## Abstract

Among the different mechanisms involved in oxidative stress, protein carbonylation and lipid peroxidation are both important modifications associated with the pathogenesis of several diseases, including cancer. Hematopoietic cells are particularly vulnerable to oxidative damage, as the excessive production of reactive oxygen species and associated lipid peroxidation suppress self-renewal and induce DNA damage and genomic instability, which can trigger malignancy. A richer understanding of the clinical effects of oxidative stress might improve the prognosis of these diseases and inform therapeutic strategies. The most common protein carbonylation and lipid peroxidation compounds, including hydroxynonenal, malondialdehyde, and advanced oxidation protein products, have been investigated for their potential effect on hematopoietic cells in several studies. In this review, we focus on the most important protein carbonylation and lipid peroxidation biomarkers in hematological malignancies, their role in disease development, and potential treatment implications.

## 1. Introduction

Oxidative stress can be defined as an imbalance between the production of reactive oxygen species (ROS) and the ability of the cells to detoxify them [1]. ROS production is a common consequence of aerobic metabolism, and can play a dual role in cells, being either beneficial or harmful. For instance, hydrogen peroxide is a major redox metabolite that operates in redox signaling, but when produced at high concentrations it can contribute to damage of biomolecules and trigger an inflammatory response [2]. High levels of ROS are associated with DNA fragmentation, lipid peroxidation, and/or protein carbonylation, leading to cellular dysfunction and even cell death [3]. Accordingly, cells rely on efficient antioxidant defenses provided by enzymes and metabolites to maintain low levels of ROS; for example, superoxide dismutase (SOD) and catalase (CAT) enzymes, and antioxidant molecules such as thiol antioxidants or vitamin E [3].

As oxidative stress has myriad consequences for cell fate, many analytical procedures have been established both for the produced ROS and their downstream effects on biomolecules. Although free radicals can be measured in biological samples, their quantification lacks sensitivity and specificity, and so much effort has been directed at quantifying their target products, including fragmented DNA, lipid peroxidation products (malondialdehyde (MDA) or 4-hydroxy-2,3-nonenal (HNE)) and protein carbonylation, an irreversible oxidative modification [4]. As DNA fragmentation does not directly correlate with ROS levels, the most useful current methods involve the quantification of lipid peroxidation and protein carbonylation [5].

Protein carbonylation is one of the most common oxidative modifications. Oxidation of proteins is of particular concern since it leads to aggregation, polymerization, unfolding, or conformational changes that may confer a loss of structural or functional activity. Oxidized protein aggregates are not readily degraded in the cell, and their accumulation causes cell dysfunction [6,7]. While many different types of protein oxidative modifications are possible, most involve protein carbonyls (aldehydes and ketones) [8]. As carbonyl groups are chemically stable, they are extremely useful for laboratory analysis, although their study is methodologically complex. The carbonyl contents of individual proteins may be assessed through derivatization of the carbonyl group with dinitrophenylhydrazine (DNPH), which forms a stable dinitrophenylhydrazone (DNP) product that can be analyzed spectrometrically or by immunoblotting [9,10]. Carbonylation research is characterized by the application of numerous protocols and proteomics workflows, which allows the measurement of compounds by several techniques. Protein carbonylation is a major final by-product of multiple oxidation pathways that occur in the cell and thus, this makes it an appropriate marker of oxidative stress [11]. In contrast, some protein modifications might just represent cellular antioxidation mechanisms as part of the oxidation-defense system [12]. To deepen into the biological implication of protein carbonylation, it is important to characterize each specific modification.

Protein carbonylation can be induced directly by the action of oxidative stress or indirectly by reactions of secondary by-products. 

**Figure 1:**
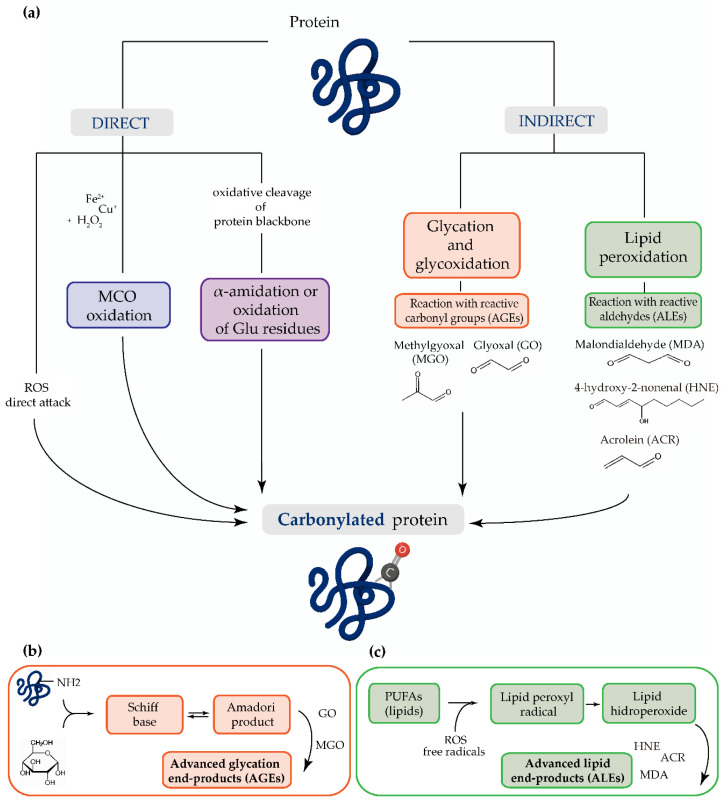
(**a**) The most common mechanisms of protein carbonylation. Direct processes include reactive oxygen species (ROS) attack, metal-catalyzed oxidation (MCO), and by oxidative cleavage of protein backbone (via the α-amidation pathway or through oxidation of glutamine side chains). The indirect mechanisms involve the reaction with (**b**) advanced glycation end-products (AGEs) and (**c**) advanced lipid peroxidation end-products (ALEs).

The main mechanism of protein carbonylation involves the direct action of ROS or the metal-catalyzed oxidation of amino acid side chains, particularly proline, arginine, lysine, and threonine. Carbonyl derivatives can also be generated through the α-amidation pathway or through the oxidation of glutamyl side chains, where the peptide is blocked in N-terminal amino acids by an α-ketoacyl derivative [13]. The indirect mechanism of protein carbonylation involves the carbonylation of lysine, cysteine, and histidine, which may be caused by their reaction with reactive carbonyl groups produced during the oxidation of carbohydrates (e.g., glyoxal (GO), methylglyoxal (MGO)) and lipids (e.g., HNE, MDA or acrolein (ACR)). This process of carbonyl generation is termed glycoxidation (the formation of advanced glycation end-products (AGEs)) and lipoxidation (the formation of ALEs), respectively [13,14,15,16,17,18]. Advanced oxidation protein products (AOPPs) are modified structures, similar to AGEs, which also serve as oxidative stress markers [19] (Figure 1).

Lipid peroxidation is also a widely used biomarker of oxidative stress. The polyunsaturated fatty acyls (PUFAs) chains found in membranes and lipoproteins are particularly susceptible to free radical chain autoxidation, leading to a variety of unsaturated lipid hydroperoxides [20]. PUFAs may also be enzymatically oxidized, although these are regio- and stereo-controlled processes involved in normal intermediary metabolism [20]. The nonenzymatic lipoxidation-derived hydroperoxides can decompose, usually in the presence of reduced metals or ascorbate [21], to generate mono- and bifunctional reactive carbonyl-containing moieties, producing aldehydes such as MDA, GO, ACR, 4-HNE, and 4-oxo-2-nonenal (ONE) [20]. The reaction of MDA with thiobarbituric acid to form thiobarbituric reactive substances (TBARS) is a common estimator of oxidative damage. The TBARS assay is, however, nonspecific for MDA, and fatty peroxide-derived decomposition products other than MDA are thiobarbituric acid-positive [20].

The potential effects of ROS as well as their target products on hematopoietic cells are particularly relevant, as these cells are acutely sensitive to oxidative damage associated with the accumulation of free radicals [22]. The resultant lipid peroxidation produced by the excessive production of ROS and reactive nitrogen species can suppress self-renewal, limiting the number of hematopoietic stem cells, and directly induce DNA damage and genomic instability [22].

In this review, we focus on the biological role of protein carbonylation-derived adducts and lipid peroxidation in hematological malignancies. A better understanding of the clinical effects of oxidative stress might improve the prognosis of these diseases and inform therapeutic strategies. Details about the main studies of these biomarkers in hematological malignancies and their treatment are summarized in Table 1 and Table 2.

## 2. Lymphoma

Lymphoma are a heterogenous group of hematological malignancies derived from different types of lymphocytes and occur predominantly in lymph nodes or other lymphoid structures [56]; as such, they are considered as the solid tumors of the immune system [57]. While their etiology is not well understood, it is likely multifactorial as abnormal genetic alterations, disordered epigenetic regulation, aberrant pathway activation, or infections like Epstein–Barr virus have been reported [56,58,59].

There is growing evidence that oxidative stress and an imbalance in reduction–oxidation might play a significant role in lymphoma carcinogenesis and patient prognosis, either by generating a more favorable environment for cancer cells to proliferate or by modifying the efficiency of oncological treatments, which are largely based on the generation of ROS [60]. Hypoxia is a characteristic feature of solid tumors and, accordingly, its role in hematological malignancies was initially presumed to be inconsequential [61,62]. As mentioned above, however, lymphoma present with solid tumor-like features [63] and normal lymph nodes exhibit low oxygen tension [60,64]. Thus, ROS production might be induced in lymphoma by hypoxic stress, whereas in other hematological diseases it might be due to impaired antioxidant defenses [65].

### 2.1. Hodgkin Lymphoma

Hodgkin lymphoma (HL) is a relatively uncommon B-cell lymphoid malignancy [66] characterized by cancerous Reed–Sternberg cells within a background of inflammation [58]. Abnormalities in oxidative–antioxidative balance have been evaluated in HL [67], and significant oxidative stress has been detected both in reactive cellular infiltrates and in cancerous cells [60], promoting senescence [68].

Several studies have analyzed the status and expression levels of antioxidant enzymes in HL, but results are conflicting, likely because the cellular antioxidative status depends on the stage of malignancy and on the histological pattern of the tumor [23,46,69,70,71]. Nevertheless, more recent studies on HL concur with an increase in the expression of the antioxidant protein thioredoxin (Trx), which plays pivotal roles in regulating multiple cellular redox signaling pathways [72] in this disease [73,74]. An increase in Trx levels in mice directly correlates with greater protection against protein carbonylation and lipid peroxidation [75] and DNA microarray-based analysis has shown that HL shows frequent losses in *TXNIP/VDUP1* [76], which encodes a protein that may act as an oxidative stress mediator through Trx inhibition [77].

**Figure 2:**
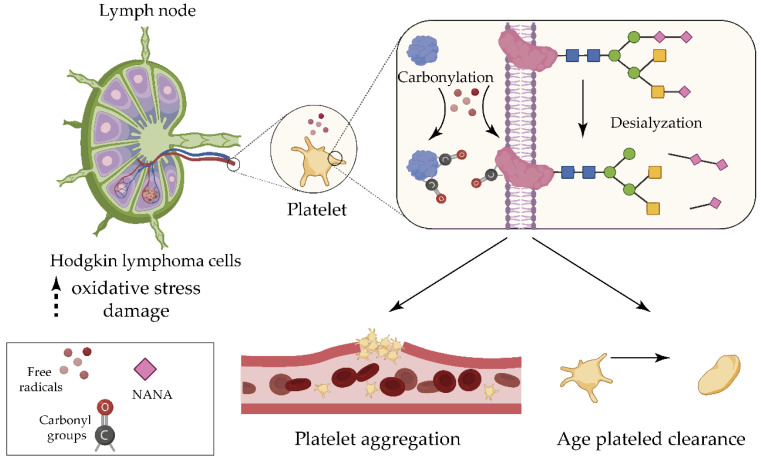
Protein carbonylation can induce membrane glycoprotein desialylation in platelets of patients with Hodgkin lymphoma. The level of sialic acid residues in platelets was found to be significantly lower and the carbonylation of proteins was higher in 10 cases of Hodgkin lymphoma compared with healthy controls [78]. A positive correlation was observed between the carbonylation of platelet proteins and the reduction in the content of N-acetyl neuraminic acid (NANA, a sialic acid residue) in platelet membrane glycoproteins, whose removal is involved in different physiological processes. The role of protein carbonylation in desialytation remains to be elucidated.

The serum content of MDA/HNE and protein carbonyl groups, is elevated in HL [14,23]. In the context of oxidant-induced injury in HL, protein carbonylation triggers desialylation of membrane glycoproteins in platelets, as evidenced by a decrease in sialic acid content of platelet proteins, which is a physiological process involved in elimination of aged glycoproteins and modulates platelet aggregation [78,79,80]. This mechanism may occur in other cells in HL, where the increase in desialylation can cause functional alteration to glycoproteins, for example promoting immune suppression or changing the cellular adhesion property, leading to cancer cell progression [78,81,82] (Figure 2).

### 2.2. Non-Hodgkin Lymphoma

Lymphoma other than HL are covered by the general term non-Hodgkin lymphoma (NHL), which again comprises a heterogeneous group of B-cell and T-cell neoplasms [83,84]. Despite the evidence that impaired antioxidative capacity may influence NHL risk [85,86,87], there are very few reports on antioxidant enzyme abnormalities, protein carbonylation, or lipid peroxidation in patients with NHL [24,25,26]. All the reports, however, conclude that serum protein carbonyl groups and MDA concentration are significantly higher in patients with NHL than in controls [24,25,26], suggesting a state of lipid and protein oxidation.

In addition to protecting against carbonylation, Trx is an activator of nuclear factor κB (NF-κB), which, among its many functions, inhibits apoptosis and stimulates proliferation [88,89]. In diffuse large B-cell lymphoma (DLBCL), which is the most common B-cell NHL [90], strong Trx expression is associated with poor progression-free survival and poor disease-specific survival [89]. Consistent with this, TXNIP/VDUP1 expression is significantly decreased in patients with DLBCL with the worst prognosis [91]. Thus, Trx appears to play a key role in cell growth and survival, as well as chemoresistance, and is a potential target to overcome drug resistance in relapsed/refractory DLBCL [92].

This body of evidence suggesting that protein carbonylation is also increased in B-cell NHL has been recently confirmed by Haddouche et al. in the serum of patients with DLBCL and Burkitt lymphoma [26].

More attention has been paid to the potential alterations in redox balance in lymphoma in response to chemotherapy. For instance, Bottari et al. observed that TBARS and AOPPs serum levels in dogs with NHL were higher after cyclophosphamide, vincristine, doxorubicin, and prednisone (CHOP) chemotherapy than before treatment; a sign of treatment-exacerbated oxidative stress caused by CHOP-related ROS generation [48]. In another study, the serum levels of MDA in NHL patients were significantly lower after CHOP treatment, leading the authors to suggest that the cytotoxic regimen destroys the oxidant/antioxidant equilibrium in serum [25]. In this context, the use of serum MDA content as a prognostic factor for NHL response to CHOP chemotherapy has been proposed because in the pretreatment context patients achieving complete remission show higher levels of MDA than nonremission patients [24]. By contrast, HL patients treated with adriamycin, bleomycin, vinblastine, and dacarbazine (ABVD) regimen showed higher MDA plasma levels after chemotherapy [46].

Furthermore, plasma L-ascorbic acid concentrations have been found reduced both in patients with HL and NHL after treatment [47]. Ascorbic acid promotes detoxification and elimination of HNE and prevents the formation of protein-HNE adducts, inhibiting protein carbonylation [15].

In summary, the fact that these hematological neoplasms have certain characteristics of solid tumors [63] has enhanced the study of the role of oxidative stress. However, the specific analysis of protein carbonylation and lipoxidation in lymphoma has been poorly described, and mainly for the assessment of potential biomarkers. However, its potential utility could be affected by the inconsistent results obtained to date. In consequence, a greater effort would be necessary to study these biomarkers in correlation with other parameters, such as molecular patterns of the disease or histological subtypes. Moreover, the presented evidence suggests that they are also involved in important physiological processes associated with the disease and its treatments. Therefore, it is essential to continue studying and elucidating the molecular mechanisms by which they act.

## 3. Multiple Myeloma

Multiple myeloma (MM) is the second most common hematological cancer after lymphoma and is characterized by the accumulation of clonal malignant plasma cells in the bone marrow (BM). In fact, the BM microenvironment (niche) plays a key role in supporting tumor cell growth, disease progression, and drug resistance of myeloma plasma cells [93]. One of the main causes of MM is indeed oxidative stress, and it has been known for almost three decades that oxidant/antioxidant parameters are misbalanced in this disease [94] which might continuously stimulate an inflammatory milieu at the tumor microenvironment [29]. The enhanced oxidative state, in turn, increases the rate of genetic mutation, leading to the acquisition of a malignant phenotype and subsequent cancer progression, as also observed in HL [67].

Commonly, MM is preceded by asymptomatic premalignant stages including monoclonal gammopathy of uncertain significance (MGUS) and/or a symptomatic stage such as smoldering multiple myeloma (SMM). Important proteins for the progression of MM, such as c-MYC, have been shown to regulate ROS levels through the modulation of mitochondrial activity [95]. Myeloma cells increase their metabolic demand when the disease progresses, and, consequently, there is a disproportionate production of free radicals or ROS.

To avoid the potential damage of the malignant cells, a heavy dependence on antioxidants is required. Thus, in MM the Trx system is promoted to maintain the redox homeostasis in protecting against their high intrinsic oxidative stress for survival and growth [32,95,96,97,98]. Besides, it is accompanied by high basal expression of Trx1 and thioredoxin reductase 1 proteins [95,96,99]. However, other antioxidant systems SOD, CAT, and glutathione peroxidase (GPX) present decreased expression, ultimately leading to changes in some signaling pathways [27,100].

In the last two decades, several studies have investigated serum markers of lipid peroxidation and protein carbonylation to address a correlation with the progression of MM disease. Circulating MDA and protein carbonyl groups were found to be increased in a well-nourished MM patient cohort [27]. However, to date no correlation was found between serum levels of carbonylated proteins and clinical markers for survival in MM [29,32].

Other markers of oxidative stress, such as AGEs or AOPPs and protein nitrosylation have been assessed in MM pathogenesis. Gangemi et al. found a significant increase in the expression of AOPPs and nitrosylated proteins in patients with MM at diagnosis as compared with patients with MGUS and controls [29]. Of note, the authors also performed a subanalysis of patients with MM and bone lesions and found that not only were AOPPs and nitrosylated proteins increased, but also a significant increase of AGEs was noted when compared with patients without bone lesions. This fits well with the notion that AGEs are involved in the pathogenesis of bone destructive alterations in MM [29]. In addition to serum, Katz et al. found that AGEs present in saliva are significantly increased in patients with MM and bone lesions as compared with peers without bone lesions [31]. In this context, several studies have investigated the AGE/RAGE axis, which stimulation contributes to the establishment of a protumorigenic microenvironment.

Somewhat controversially, Allegra et al. found that serum AGEs are lower in patients with MM than in controls [33]. Surprisingly, however, the expression of RAGE was significantly higher in patients with MM. It is feasible that the difference in AGE levels might be influenced by the fluctuating RAGEs expression levels. By investigating the elevations in RAGE, they later demonstrated that activation of RAGE modified intracellular signals to stimulate NF-κB, which in turn modified gene expression and increased ROS levels. RAGE is a multiligand transmembrane receptor that can be expressed on various cell types. Moreover, one of its forms, soluble RAGE (sRAGE) is found in circulation, easily detected by ELISA. In MM patients the increase in sRAGE is proposed to confer protection by reducing the negative action exerted by AGEs [101]. It has been suggested sRAGE acts as a ligand decoy, avoiding AGE/RAGE binding and consequent damage, being useful as a novel therapeutic approach [101,102].

Similar to the observations in patients with lymphoma, oxidative stress parameters can be altered as a consequence of chemotherapeutic agents. Former regimens, such as vincristine, adriamycin and dexamethasone (VAD) scheme, have been assessed for MDA plasma levels revealing a significant reduction compared to patient samples at diagnosis [49]. However, there is a lack of studies analyzing these parameters’ status with current therapies. Although Mehdi et al. described a significant reduction of AOPPs and MDA levels after 1-month induction therapy, unfortunately there is no information about the drugs used [50].

Nowadays, to note that current treatments produce important side effects that are caused by the formation of adducts with drugs such as bortezomib [103,104], carfilzomib [105], or melphalan [106]. Bortezomib is a proteasome inhibitor as well as carfilzomib, and one of the first-line drugs to treat MM. Patients receiving bortezomib have been shown to increase the abundance of MGO, a major precursor in the formation of AGEs and a contributor to the neuropathic pain induced by bortezomib [107]. Additionally, it was found that MGO activates the RAGE pathway, which contributes to central sensitization and allodynia [108]. Likewise, Karademir et al. showed that bortezomib causes high protein carbonylation and ubiquitinated-protein accumulation in neural stem cells, which could explain its associated neurotoxicity. Additionally, cardiomyoblastic cells exposed 24 h to carfilzomib showed also high protein carbonylation and ubiquitinated-protein accumulation, supporting the reported cardiotoxic side effects of carfilzomib in the clinic [107]. In a rat study, carfilzomib treatment resulted in a significant increase in heart MDA content, accompanied by a decrease in cardiac glutathione (GSH) levels and CAT enzyme activity. They found these alterations were reversed by treatment with the PDE4 inhibitor apremilast, suggesting carfilzomib in combination with compounds that avoid the stimulation of oxidative stress may represent a good strategy to escape to the carfilzomib side effects [109]. Elevated carbonylation of cardiac myosin-binding protein has been observed after doxorubicin treatment, which also could explain its adverse cardiac effects [110].

To sum up, it is clear that malignancy progression occurs in MM as oxidative stress increases, while changes in the microenvironment encourage metabolism reprogramming on plasma cells. Controversial results, like discrepancy in AGE levels are commonly found, revealing a current lack of protein carbonylation and lipid peroxidation biomarkers characterization in MM patients. A more detailed analysis of the AGE/RAGE status could shed light on this controversy. It is important to increase the knowledge of the oxidant/antioxidant status in the different stages of the disease and their treatment responses to achieve a characterization leading to mitigate the undesired effects of oxidative stress.

## 4. Leukemia

Leukemia is known to be a heterogeneous disease and four major subtypes are recognized: acute lymphoblastic leukemia (ALL), chronic lymphoblastic leukemia (CLL), acute myeloid leukemia (AML), and chronic myeloid leukemia (CML). ALL is the most frequent cause of death from cancer before the age of 20 [111] and presents genomic alterations implicated in the proliferation and maturation of lymphoid progenitor cells [112]. CLL is the most common leukemia worldwide and is characterized by the accumulation of B (B-CLL) or T (T-CLL) cells arrested in the early phase of cell division [38], although its ontogeny is unknown [113]. AML is the most common acute leukemia in adults and is defined by the clonal expansion of abnormally differentiated blasts of the myeloid lineage [114]. Finally, CML is a myeloproliferative disorder with a unique genetic rearrangement, the *Philadelphia chromosome* (BCR-ABL1) which causes the disease [115]. 

Oxidative stress is a prominent feature in many leukemias [4], and they are characterized by a higher level of ROS than nonleukemic cells. The implication of ROS in the course of leukemias has been scarcely studied [34,65,116,117].

### 4.1. Acute Lymphoblastic Leukemia

In a study of 80 children with ALL, Battisti et al. found that the plasma levels of reactive TBARS and the serum levels of protein carbonyls were significantly higher in patients than in controls, and was accompanied by alterations in enzymatic antioxidant defenses, where SOD and CAT antioxidant activities were significantly reduced at diagnosis moment [3]. However, they found no differences in oxidative levels between patients just diagnosed and those under treatment or after therapy in a subgroup analysis, and so the increase in the oxidative stress is likely not a result of the treatment but may be involved in the pathogenesis of the disease. 

### 4.2. Chronic Lymphoblastic Leukemia

Studies of oxidative stress in CLL are mainly focused in the study of changes in B-CLL and are controversial. Musolino et al. found that patients with B-CLL have higher serum levels of carbonyl groups than controls, and also that these levels correlate positively with CD38 expression and negatively with ZAP70 expression (both markers of poor outcome), proposing them as a prognostic factor in B-CLL [36]. In a similar study, Gangemi et al. analyzed the serum levels of AOPPs and AGEs, and protein nitrosylation in patients with B-CLL, and found that whereas AOPPs, AGEs, and S-nitrosylated proteins are increased in patients, there is no correlation between any of these products and CD38 or ZAP70 expression, nor with mutations in the IgVH gene [19]. With respect to lipid peroxidation, several studies show that MDA levels are increased in B-CLL, both in plasma [37] and serum [38], and further increase in parallel with the evolution of the disease [38,118], or are related to unfavorable cytogenetic aberrations [119], although this is not always the case [34].

### 4.3. Acute Myeloid Leukemia

In a study of 49 patients with leukemia, including AML, the measured levels of plasma MDA were found to be within the normal range [35], likely as a result of the increased protective response of antioxidant enzymes; although a clear increase in ROS production has also been described [35,116]. Despite the lack of studies on protein carbonylation in AML, it has been described that HNE increases the formation of protein carbonyl radicals in THP-1 cells [15]. Increased levels of oxidative stress markers and impaired antioxidant capacity have been described, but only in patients with AML when accompanied by depression [120]. This suggests that oxidative stress plays a role in the pathophysiology of depression in AML, which has been successfully modeled in rats [121].

### 4.4. Chronic Myeloid Leukemia

Higher plasma levels of protein carbonylation and lipid peroxidation have been observed in patients with CML when compared with healthy controls [1,4,5,39]. Additionally, it has been shown that the levels of oxidative products levels seem to be higher in patients who progress from the chronic to the accelerated phase than in patients who do not progress [5,39].

As we have mentioned earlier, chemotherapeutic agents generate enormous amounts of free radicals associated with cell injury. Doxorubicin-transferrin conjugate treatment of human ALL and CLL cell lines stimulates high levels of ROS and of thiols and carbonyl groups [51]. Other studies in acute promyelocytic leukemia (APL) cell lines show that the levels of lipid peroxidation are higher after cisplatin [16,53] or arsenic trioxide treatment [54,55]. Esfahani et al. followed the oxidant status of patients with AML before and after 14 days of chemotherapy with daunorubicin and cytarabine and found that lipid peroxidation levels increased after treatment whereas antioxidant enzymes levels decreased [52]. With respect to B-CLL, a correlation between oxidative stress and drug resistance has been proposed based on the expression of multidrug resistance gene polymorphisms [19].

In summary, there are very few articles investigating oxidative stress in leukemia. Most of them describe an increase in ROS production that could translate into an increase in protein carbonylation and lipid peroxidation. This increase is clearly detected in chronic leukemias [1,4,5,19,34,36,37,38,39,118,119], but it is controversial in acute leukemias, where the levels of antioxidant defense mechanisms seem to play a critical role [3,35,52,120]. Increased levels of protein carbonylation and lipid peroxidation have been detected in ALL together with the reduction of the antioxidant activity of SOD and CAT [3]. The opposite situation occurs in AML, where lipid peroxidation levels are within the normal range, but accompanied by an increase in the protective response of antioxidant enzymes SOD and GPX [35]. Enhanced oxidative stress biomarkers were only detected in AML associated with depression [120] or after chemotherapy [52], with an evident impairment of antioxidant capacity in both cases [52,120]. Then, in order to study the implication of protein carbonylation and lipid peroxidation in leukemia, it is important to correlate it with the antioxidant defense status, especially in acute leukemias. Of interest, some studies describe that ROS increase along the evolution of the disease, specifically in chronic leukemias [5,38,39,118], but this awaits study in acute leukemias. Accordingly, more studies are needed to better understand the specific role of oxidative stress in leukemias, whether it could be used as a prognostic marker or as a druggable target.

## 5. Myelodysplastic Syndromes

Myelodysplastic syndromes (MDS) are also a heterogeneous group of onco-hematological cell disorders that are characterized by the presence of immature myeloid precursors (blasts), dysplastic hematopoiesis in the BM, and peripheral cytopenias [122,123,124]. Approximately one-third of MDS cases progress to AML [125,126]. 

**Figure 3:**
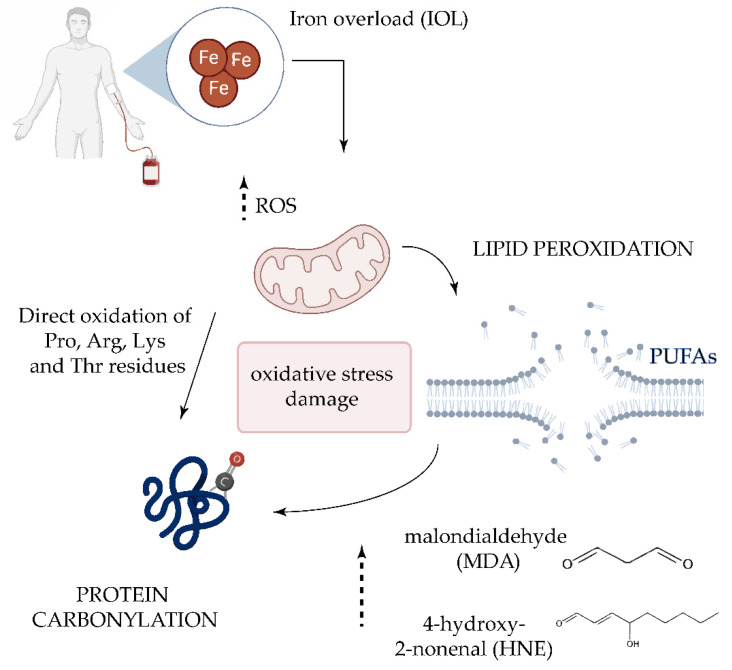
Oxidative damage in transfusion-dependent patients with MDS. Frequent transfusions in patients with MDS leads to iron overload in serum, which plays a key role in the generation of highly reactive oxygen species (ROS) [135]. The increase of ROS could directly oxidize proline (Pro), Arginine (Arg), Lysine (Lys), and Threonine (Thr) residues. ROS-induced lipid peroxidation of long-chain polyunsaturated fatty acids (PUFAs) also promotes protein carbonylation by reaction with lipid peroxidation end-products such as malondialdehyde (MDA) and 4-hydroxy-2-nonenal (HNE).

Although its origin is not well understood, the role of oxidative stress in the pathogenesis of the MDS have been investigated in several studies [122,127,128,129]. Approximately 60–80% of patients experience symptomatic anemia and 80–90% require red blood cell transfusion support [130]. For this reason, many patients with MDS develop transfusion-dependent iron overload (IOL) [131]. When present in excess, cellular iron leads to toxicity and cell death via free radical formation and lipid peroxidation [132]. It has been reported that the development of IOL significantly worsens the survival of patients with MDS, and it is associated with a higher risk of leukemic transformation [133]. While the role of ROS in MDS is established, the role played by lipoxidation products in the disease and its progression is unclear [9]. High concentrations of the HNE adduct have significant cytotoxic effects on DNA synthesis and mitochondrial activity in leukemic cells, but not in normal hematopoietic precursor cells [134]. However, a recent study examining protein carbonylation in MDS found no significant differences in HNE adducts in BM samples between the MDS and the control group [44]. Analysis of the lipid peroxidation products MDA and nitrite revealed significantly higher levels in patients with MDS and IOL compared with peers without IOL and the control group, and both parameters positively correlated with the levels of ferritin [41] (Figure 3). The same authors later confirmed the increase in MDA levels and higher levels of antioxidant enzymes [22], suggesting that increases in lipid peroxidation is followed by an increase in antioxidant capacity.

Regarding the concentration of carbonyl groups produced in proteins as a tool for understanding the role of oxidative stress in this disease [136], in a study on 32 patients of different MDS subgroups, Hlaváčková et al. found significant differences in the carbonylation of plasma proteins between all MDS patients and healthy controls [40]. They also found significantly increased carbonyl levels in MDS with ring sideroblasts (MDS-RS) (low risk) patients, correlating with the findings of Cortelezzi et al. with nontransferrin bound iron levels, which were also higher in patients with low-risk MDS and favor oxidative DNA damage [42]. Moreover, Hlaváčková et al. identified 27 carbonylated proteins unique for MDS-RS, most of them related with the acute-phase inflammation process [40]. Consistent with these findings, an analysis of protein carbonylation in the BM of patients with MDS showed a high level of carbonylation both in myeloid series and in erythroid precursors in patients [44].

MDA levels were found to be uniformly higher in patients than in controls [42]. In contrast to these studies, Pimková et al. failed to find significant differences in plasma levels of MDA and nitrate between patients with MDS and healthy controls [43]. However, they found a correlation between MDA levels in plasma and both serum ferritin levels and serum free iron levels and MDA levels were significantly higher in patients with iron overload [43].

Regarding glycoxidation, AGEs are strongly associated with aging-related diseases, including cancer. Interestingly, although MDS is one of the most common hematologic malignancies in patients over the age of 70 years [137], there is a lack of studies regarding this pathway. 

In brief, the iron overload in patients with MDS leads to the generation of highly reactive oxygen species (ROS) and protein carbonylation, whose misbalance have an impact on the development and overall survival of the disease. The analyses of the lipid peroxidation products concur that MDA levels were higher in patients with the disease, but the involvement of the HNE is less clear. Further studies are warranted to deepen understanding of the mechanisms of the protein carbonylation and oxidative stress involved in the MDS disease.

## 6. BCR/ABL Negative Myeloproliferative Neoplasms

BCR/ABL negative myeloproliferative neoplasms (MPNs) are unique hematopoietic stem-cell disorders that share mutations that constitutively activate the physiologic signal-transduction pathways responsible for hematopoiesis [138]. MPNs are clonal disorders that are mainly characterized by hyperproliferative BM with varying degrees of reticulin/collagen fibrosis, extramedullary hematopoiesis, abnormal peripheral blood count, and constitutional symptoms. They include polycythemia vera (PV), essential thrombocythemia (ET), and primary myelofibrosis (PMF) [139]. 

An imbalanced oxidative status, higher ROS levels, and lower total antioxidant capacity levels compared with controls was found in patients with myelofibrosis by Verner et al. Additionally, the oxidative stress and MDA levels were increased, whereas the total antioxidant status was lower [140]. Following therapy, oxidative stress index and MDA values were significantly lower than the pretreatment values [141]. Higher plasma levels of MDA together with significantly higher protein carbonyls content was also recently reported in patients with MPNs compared with healthy subjects [45].

In patients with PV and ET, Musolino et al. evaluated oxidative stress, finding higher levels of advanced oxidated protein products and S-nitrosylated proteins in both diseases and an increase of AGEs in patients with ET with respect to controls. The authors found a correlation between S-nitrosylated proteins and hemoglobin values in patients with PV, and between AGEs and thrombotic events in patients with ET, suggesting a potential role of ROS in the onset of myeloproliferative-associated thrombotic risk [142].

## 7. Oxidative Stress Modulators for the Treatment of Hematological Malignancies

**Figure 4:**
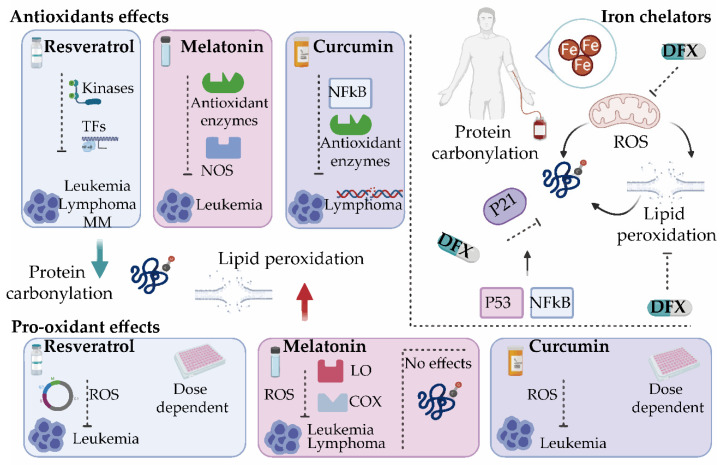
Oxidative stress modulators for the treatment of hematological malignancies. Several antioxidants such as resveratrol, melatonin, and curcumin exert both antioxidant and pro-oxidant activities. By modulating different antioxidant enzymes and transcription factors (TFs), these compounds reduce protein carbonylation and/or lipid peroxidation and inhibit tumor progression. They present cytotoxic effects by enhancing reactive oxygen species (ROS) production. Iron chelators such as deferasirox (DFX) inhibit ROS production directly or indirectly by suppressing the active redox forms of iron and regulating mitochondrial activity and can significantly decrease lipid peroxidation and protein carbonylation in a mechanism dependent on the cell cycle and p21. It will be interesting to explore whether DFX exerts its control on p21 through NF-κB [145] or by inhibiting signaling pathways activated by oxidative stress that control the cell cycle via p53 [146]. COX, cyclooxygenase; LO, lipoxygenase; MM, multiple myeloma; NOS, nitric oxide synthase.

As discussed earlier, the misbalance of ROS production in cancer cells can alter cell survival mechanisms. Accordingly, a number of studies have focused on developing new treatment strategies by targeting the redox system in tumor cells. Interestingly, while some of the tested agents exert antioxidant properties, a large number of them are also documented to increase the levels of intracellular ROS in addition to blocking a biochemical target [143,144]. Given the large number of targeted therapies tested, we focused only on those agents that have a modulatory effect on oxidative stress, by affecting either protein carbonylation or lipid peroxidation (Figure 4).

### 7.1. Potential Antioxidant Drugs

As the total cellular antioxidant capacity is generally compromised in hematologic malignancies, administration of antioxidant drugs might represent a successful way to restore the redox balance. Several antioxidants have been already evaluated as potent anticarcinogenic agents in different kinds of tumors, both in monotherapy or in combination with other antioxidants or classical chemotherapeutics and some of them even revealed promising results in clinical trials. Compared to classical treatment, these compounds possess several important advantages. Beside lower costs they do not exert serious side effects on normal tissues and can be used for chemoprevention [144].

Antioxidants, such as spirulina [147], *Enhydra fluctuans* extracts [148], and selenium [149,150] can re-establish hematological parameters while simultaneously reducing protein carbonylation and/or lipid peroxidation levels. 

In the context of hematological tumors, the antitumor activity of the natural polyphenol resveratrol has been tested in virtually all types of blood cancer cells, including leukemias, lymphomas, and MM [151,152,153,154]. Resveratrol protects phospholipids from oxidation [144], although it is able to inhibit all stage of carcinogenesis (e.g., initiation, promotion, and progression) [155,156,157] by modulating transcription factors, upstream kinases, and their regulators [158]. As resveratrol has no impact on hematopoiesis [159] its potential utility for ex vivo pharmacological purging of leukemia cells from BM autografts before transplantation has been proposed [160,161]. Resveratrol has also been shown to reverse drug resistance in a broad range of in vitro cell systems by sensitizing tumor cells to drug-mediated effects in combination with other chemotherapeutic agents [156]. Nevertheless, a phase 2 clinical trial of resveratrol with or without bortezomib for patients with relapsed and/or refractory MM highlighted side effects and an unacceptable safety profile in combination with bortezomib in these patients [162]. 

The pituitary hormone melatonin (N-acetyl-5-methoxytryptamine) is of great interest as an endogenous redox modulator with anticancer activity. Melatonin acts directly as a chelator of ROS [163,164,165,166,167], and indirectly by regulating the expression and activities of antioxidant enzymes and nitric oxide synthase [168,169,170,171]. Melatonin has been shown to have a synergistic cytotoxicity effect in combination with lymphoblastic leukemia drugs such as doxorubicin, decreasing ROS and carbonyl formation. Interestingly, this combination is safe for normal lymphocytes, pointing to melatonin as a promising adjuvant for anticancer therapy by allowing lower doses of the anticancer drugs, minimizing their side-effects [172].

In in vitro models of lymphoma, a reduction in lipid peroxidation and/or protein carbonylation products have also been reported using a variety of antioxidants, including 2,2,6,6-tetramethylpiperidin-1-oxyl (TEMPO), ascorbic acid (vitamin C), α-tocopherol, β-carotene [173], flavonols such as quercetin and rutin [174], canthaxanthin [175] or mangiferin, a C-glycosyl xanthone [176]. Interestingly, extracts of *Cocculus hirsutus* [177], β-carotene [178], and α-Tocopherol [179] increased survival time in vivo.

In addition, in a murine lymphoma model, ellagic acid inhibits lipid peroxidation and protein carbonylation, decreases PKCα c-Myc expression, and improves TGF-β1 expression in addition to decreasing cell viability, supporting its anticarcinogenic action [180,181].

Another natural product belonging to the group of polyphenols is curcumin or diferuloylmethane, which inhibits free radicals from mediating peroxidation of membrane lipids or oxidative DNA damage [144]. The chemotherapeutic potential of curcumin has been also tested in mice with lymphoma. Results showed that curcumin administration leads to a decrease in lipid peroxidation and protein carbonylation levels, and an increase in the expression and activity of antioxidant enzymes, which in turn modulate the activation of NF-κB, overall reducing lymphoma growth [182].

In conclusion, antioxidants are promising drugs in the management of hematological malignancies. Nevertheless, further studies are necessary in order to confirm their role as anticancer compounds. It should be noted that there are conflicting opinions on the administration of antioxidants during cancer therapy. It is still largely unaccepted by the clinical community as some oncologists believe that it may reduce the effectiveness of chemotherapies, which are mostly based on increasing oxidative stress [67].

### 7.2. Potential Pro-Oxidant Drugs

In addition to antioxidants, several pro-oxidant drugs capable of modulating cellular ROS contents are currently being tested for their possible use in hematological malignancies. Some of them exert both antioxidant and pro-oxidant properties. Remarkably, the aforementioned antioxidants, melatonin, curcumin, and resveratrol, have also been described as potent pro-oxidants in cancer treatment [144,183,184].

Resveratrol has also dose-dependent pro-oxidant effects, measured as protein carbonylation, which is followed by apoptosis and cell damage [185]. Likewise, Gautam et al. demonstrated that resveratrol induces apoptotic DNA fragmentation in three leukemia cell lines (32Dp210, L1210, HL-60) but not in normal BM cells [161]. The pro-oxidant activity of resveratrol has also been linked to the induction of cell cycle arrest [186]. Resveratrol also suppresses growth of myeloid cells. Lee et al. demonstrated that resveratrol inhibits proliferation of promyelocytic leukemia cells and nonmalignant B-cell lymphoblastoid cells by blocking cell cycle progression in G0/G1, and also induces apoptosis in promyelocytic leukemia cells and acute lymphocytic leukemia cells [187,188]. Interestingly, leukemic lymphoblasts isolated from pediatric patients with ALL undergo apoptosis when treated with resveratrol [144,189].

Melatonin seems to stimulate the production of ROS in human myeloid HL-60 cells, eliciting cytotoxic effects [190]. It also increases the activity of lipoxygenases and cyclooxygenase and promotes the production of ROS in Burkitt lymphoma BL41 cells [191]. Similarly, it enhances cell death in Jurkat leukemia cells via a pro-oxidant pathway [192]. When used on tumoral leukocytes, melatonin produces a rapid and transient stimulation of intracellular ROS, but does not lead to oxidative stress, as revealed by absence of protein carbonylation and the maintenance of free thiols [92,193].

Curcumin also shows pro-oxidant anticarcinogenic mechanisms that are concentration dependent: whereas low concentrations decrease ROS production in human leukemia cells, higher concentrations have the opposite effect and favor ROS generation, measured as increased MDA levels [194]. Curcumin exerts cytotoxic activity on human T-cell leukemia cells without affecting normal cells [195]. Mechanistically, curcumin seems to affect histone acetyltransferase [196] and thioredoxin reductase, converting the latter into a pro-oxidant [197]. Moreover, curcumin induces an increase in GSH levels, responsible for the induction of an apoptotic death pathway in lymphoid Jurkat cells [144,198].

Despite the promising anticancer effects of these natural products, contradictory results responsible for its dual effects are continuously described [199]. Chemically unstable structure, feeble pharmacokinetic, and low bioavailability are reasons for the broad bioactivity profile of these compounds, blocking them from reaching maturity as a drug lead. Moreover, the potential health benefits are still questioned. It is important to highlight there is an urgent need to better characterize the polypharmacology of their degradation products. The lack of chemical standardization of potential drugs like resveratrol and curcumin limits an adequate control of biological assays, leading to unpredictable or potentially irreproducible results [200,201].

A novel redox active mediator that selectively targets tumor cells is motexafin gadolinium, which is a synthetic compound that directly inhibits the activity of Trx and protects against protein carbonylation and lipid peroxidation by inducing apoptosis in malignant cells through oxidative stress. Motexafin gadolinium is being tested clinically for the treatment of lymphoma (NCT00089284, NCT00086034) and leukemia [75,202,203].

Arsenic trioxide is used successfully for the treatment of APL, and both induction and consolidated therapy have resulted in complete remission. Kumar et al. demonstrated that arsenic trioxide induces significant oxidative stress (lipid peroxidation), DNA damage, and caspase 3 activity in HL-60 cells in a dose-dependent manner, and reduces GSH levels [54]. It also activates the intrinsic pathway of apoptosis by modulating the translocation of apoptotic molecules such as Bax and cytochrome c and decreasing the mitochondrial membrane potential.

Finally, targeting copper in cancer cells can also serve as an effective anticancer strategy. Copper is an important metal ion associated with the chromatin DNA. Unlike normal cells, cancer cells have elevated copper levels, which play an integral role in angiogenesis. The interaction between Cu(II) and the phytoestrogen coumestrol in lymphocytes results in lipid peroxidation, protein carbonylation, DNA fragmentation, and apoptosis [204].

These data reinforce the necessity of studying the role of oxidative stress modulating compounds in hematological tumors, characterizing not only their pro/antioxidant effects, but also their molecular mechanism. Although the anticancer potential of some natural products is controversial, some compounds (e.g., motexafin gadolinium) present promising results. Moreover, the clinical efficacy of these agents would be assessed by using biomarkers such as carbonylation and lipid peroxidation.

### 7.3. Iron Chelators

Iron homeostasis is an effective target in the treatment of different hematological tumors, particularly MDS. As discussed earlier, most patients with MDS develop transfusion dependence and IOL, which has a negative impact increasing oxidative stress parameters [9,128,205,206,207]. In this setting, iron chelation, mainly by Deferasirox (DFX) appears to improve survival in patients with lower-risk MDS and in stem cell transplant settings [206,208]. Moreover, it has been shown to reduce mortality and cytopenia and improve the hematological response [9,209,210,211,212].

Deferasirox is an iron chelator commonly used as a treatment in patients with MDS relying on blood transfusions [213]. DFX is a powerful NF-κB inhibitor in myelodysplastic cells acting independently of cell iron deprivation by chelation, and ROS scavenging [145] and the inhibition of the de novo generation of free radicals through the suppression of the active redox forms of iron [135]. DFX constrains ROS damage in hematopoietic progenitor cells by activating transcription factors and mitochondrial biogenesis [214], the dysfunction of which has been observed in cases of MDS with IOL [215]. Interestingly, a significant decrease in the mean levels of ROS and membrane lipid peroxidation has been reported during DFX therapy [216,217]. In addition, patients under DFX treatment have lower levels of protein carbonylation in BM with respect to untreated patients, which is accompanied by a reduction in the expression of the p53 target gene, p21 [44]. It would be interesting to explore whether DFX exerts its control on p21 through NF-κB [145] or, alternatively, by inhibiting signaling pathways activated by oxidative stress that control the cell cycle via p53 [44,146].

Despite the paucity of studies investigating the beneficial effects of iron chelation on protein carbonylation in other hematological diseases, its effect on ROS levels, and thus, on disease control seem robust.

Iron chelating therapy in myeloid leukemias induces the differentiation of leukemia blasts and normal BM precursors into monocytes/macrophages, in a manner involving the modulation of ROS expression [218]. Moreover, the cytotoxic effects of iron chelating therapy on myeloid blasts has been reported in vitro, in vivo, and ex vivo [219,220,221,222,223,224], presenting a synergistic effect with AML drugs such as decitabine and 5-azacytidine [225,226]. In contrast to the effects of decitabine, DFX decreases the ROS levels to varying degrees [225]. Human studies have demonstrated a protective role of DFX after allogenic-hematopoietic stem cell transplantation in AML [227,228] and in a patient with chemotherapy-resistant AML [229].

DFX has also been reported to inhibit mantle cell lymphoma cell proliferation [230,231], and iron deprivation is cytotoxic to malignant B- and T-cells [232,233]. Moreover, in ALL and T-cell lymphoma, DFX displayed synergistic activity with three ALL-specific drugs: dexamethasone, doxorubicin, and L-asparaginase. Iron chelation appears to act through a ROS-dependent DNA damage response and potentiates the action of an inhibitor of the PARP pathway of DNA repair [234].

Finally, in the context of MM, chelation of intracellular iron induces cell death in myeloma cells [235]. Deferasirox also induces apoptosis in MM cells by targeting oncogenic Pyk2/β-catenin signaling [236]. Conversely, iron loading impairs cell proliferation in MM and increases the efficacy of bortezomib, as iron causes lipid oxidation and inhibits proteasome function [237].

## 8. Conclusions

There seems to be solid evidence that oxidative stress has a significant role in promoting carcinogenesis in hematological cells. Here, we explored the main biomarkers of oxidative stress. The bulk of the literature describes an increase in ROS production that could be translated into an increase of protein carbonylation and lipid peroxidation in patients with hematological malignancies. Despite this increase in ROS production has been described in all the hematological malignancies, there are no specific studies analyzing this implication in the disease’s prognosis. Although there is no prognostic classification which includes molecular markers of oxidative stress, probably they could be beneficial. In this line, the evaluation of protein adduct levels might be useful as a prognostic factor and as a marker of the development of these diseases, as well as the quantification of the serum carbonyl groups. As the levels of oxidative stress biomarkers have been correlated with the progression of some hematological malignancies, the use of different therapies targeting the imbalance in the redox system might restore protein carbonylation and/or lipid peroxidation levels and maybe avoid disease progression in certain specific patients. Consequently, antioxidant and pro-oxidant drugs might represent a successful strategy to overcome some hematological malignancies. Indeed, several studies have reported the beneficial effect of iron chelators on protein carbonylation and ROS levels as treatments for hematological malignancies. Despite some promising data in patients, there remains a lack of studies focused on the molecular mechanisms behind these changes, which are needed to open new therapeutic horizons in hematological malignancies. All studies described along this review support that more specific studies are needed in order to establish the potential use of some oxidative stress biomarkers and novel therapeutic opportunities in hematological malignancies.

## Figures and Tables

**Table 1:** Major protein carbonylation and lipid peroxidation biomarker levels identified in patients with hematological malignancies.

Malignancy	Sample Tested	N Patient	N Control	Biomarkers							Ref.
				MDA	PC	AOPPs	PN	AGEs	TBARS	4-HNE	
HL	Plasma	30	30	↑							[23]
HL	Erythrocytes	30	30	↑↑							[23]
HL	Serum	15	10	↑↑↑	↑↑↑						[14]
DLBCL	Serum	40	15	↑↑↑							[24]
NHL	Serum	146	60	↑↑↑							[25]
B-NHL	Serum	32	25	↑↑	↑↑						[26]
MM	Serum	50	50	↑↑↑							[27]
MM	Serum	21	30		↑↑↑						[28]
MM	Serum	20	23			↑↑↑	↑↑↑				[29]
MGUS	Serum	8	23			ns	ns				[29]
MM	Serum	20	20	↑↑							[30]
MM	Saliva	30	7					ns			[31]
MM	Plasma	24	20		↑↑						[32]
MM	Serum	19	16					↓↓			[33]
ALL	Plasma	80	50						↑↑↑		[3]
ALL	Serum	80	50		↑↑↑						[3]
ALL	Plasma	16	15	ns							[34]
ALL	Plasma	7	20	ns							[35]
B-CLL	Serum	48	30		↑↑↑						[36]
B-CLL	Serum	60	23		↑↑↑	↑↑↑		↑↑			[19]
B-CLL	Plasma	50	31	↑↑							[37]
B-CLL	Serum	20	15	↑							[38]
AML	Plasma	11	15	ns							[34]
AML	Plasma	34	20	ns							[35]
CML	Plasma	3	15	ns							[34]
CML	Plasma	8	20	ns							[35]
CML	Plasma	20	10	↑	↑						[4]
CML	Plasma	47	20	↑	↑						[39]
CML	Plasma	40	20		↑				↑		[1]
CML	Plasma	128	50						↑↑		[5]
MDS	Plasma	32	20		↑						[40]
MDS	Plasma	76	45	↑							[41]
MDS	Plasma	78	87	↑↑							[22]
MDS	Serum	33	10	↑↑							[42]
MDS	Plasma	61	23	ns							[43]
MDS	BM	21	13		↑↑					ns	[44]
MPN	Plasma	73	10	↑↑	↑						[45]
MPN	Serum	34	23			↑↑	↑↑↑	ns			[36]

Notes: MDA, monodicarbonyls malondialdehyde; PC, protein carbonylation; AOPPs, advanced oxidation protein products; PN, protein nitrosylated; AGEs, advanced glycation end products; TBARS, thiobarbituric acid reactive substances; 4-HNE, 4-hydroxynonenal; Ref., reference; HL, Hodgkin lymphoma; DLBCL, diffuse large B-cell lymphoma; NHL, non-Hodgkin lymphoma; MM, multiple myeloma; MGUS, monoclonal gammopathy of undetermined significance; ALL, acute lymphoblastic leukemia; B-CLL, B-cell chronic lymphoblastic leukemia; AML, acute myeloid leukemia; CML, chronic myeloid leukemia; MDS, myelodysplastic syndromes; BM, bone marrow; MPN, myeloproliferative neoplasms; ns, nonsignificant; ↑, *p* ≤ 0.05; ↑↑ or ↓↓, *p* ≤ 0.01; ↑↑↑, *p* ≤ 0.001.

**Table 2:** Major protein carbonylation and lipid peroxidation biomarker levels identified in patients with hematological malignancies or in preclinical models after chemotherapeutic treatment.

Malignancy	Chemotherapy Regimen	Measurement	N	Biomarkers					Ref.
				MDA	TBARS	AOPPs	Ascorbic Acid	Carbonyl Groups	
HL	ABVD	Plasma	34	↑↑↑					[46]
HL	CHOP	Plasma	5				↓↓↓		[47]
NHL	CHOP	Serum	146	↓↓↓					[25]
NHL	CHOP	Plasma	6				↓↓↓		[47]
NHL *	CHOP	Serum	25		↑↑↑	↑↑			[48]
MM	VAD	Plasma	14	↓					[49]
MM	IT	Plasma	30	↑↑		↓			[50]
ALL	GBTLI LLA-99 protocol	Plasma	80		ns				[3]
CML ALL	DOX-TRF	Cell lines (K562 and CCRF-CEM)	6					↑ ↑	[51]
AML	Cytarabine and daunorubicin	Plasma	38	↑					[52]
APL	Cisplatin	Cell lines (HL-60, NB4 and KG-1a)	3	↑↑					[53]
APL	Arsenic trioxide	Cell line (HL-60)	3	↑					[54]
APL **	Arsenic trioxide	Cardiac tissue	6	↑					[55]

Notes: HL, Hodgkin lymphoma; NHL, non-Hodgkin lymphoma; MM, multiple myeloma; CML, chronic myeloid leukemia; ALL, acute lymphoblastic leukemia; APL, acute promyelocytic leukemia; ABVD, adriamycin, bleomycin, vincristine and dexamethasone; CHOP, cyclophosphamide, vincristine, doxorubicin, and prednisone; VAD, vincristine–adriamycin–dexamethasone; IT, induction therapy; DOX-TRF, doxorubicin-transferrin; MDA, malondialdehyde; TBARS, thiobarbituric acid reactive substances; AOPPs, advanced oxidation protein products; Ref., reference; ns, nonsignificant; ↑ or ↓, *p* ≤ 0.05; ↑↑, *p* ≤ 0.01; ↑↑↑ or ↓↓↓, *p* ≤ 0.001. * The study was carried out in dogs with diffuse large B-cell, lymphoblastic, Burkitt-like, follicular, lymphocytic, or lymphoplasmacytic lymphoma. ** The study was carried out in male Wistar rats. We consider the experimental groups treated with 4 and 8 mg As_2_O_3_/kg body weight.

## Abbreviations

4-HNE	4-hydroxy-2,3-nonenal
ABVD	Adriamycin, Bleomycin, Vincristine and Dexamethasone
ACR	Acrolein
AGEs	Advanced glycation end-products
ALEs	Advanced lipid peroxidation end-products
ALL	Acute lymphoblastic leukemia
AML	Acute myeloid leukemia
AOPPs	Advanced oxidation protein products
APL	Acute promyelocytic leukemia
B CLL	B-cell chronic lymphoblastic leukemia
BM	Bone marrow
CAT	Catalase
CHOP	Cyclophosphamide, Vincristine, Doxorubicin, and Prednisone
CML	Chronic myeloid leukemia
COX	Cyclooxygenase
DFX	Deferasirox
DLBCL	Diffuse large B-cell lymphoma
DNP	Dinitrophenylhydrazone
DNPH	Dinitrophenylhydrazine
DOX-TRF	Doxorubicin-transferrin
ET	Essential thrombocythemia
GO	Glyoxal
GPX	Glutathione peroxidase
HL	Hodgkin lymphoma
IOL	Iron overload
LO	Lipoxygenase
MAD	Malondialdehyde
MGO	Methyglyoxal
MGUS	Monoclonal gammapathy of undetermined significance
MM	Multiple myeloma
MPN	Myeloproliferative neoplasms
NANA	N-acetyl neuraminic acid
NHL	Non-Hodgkin lymphoma
NOS	Nitric oxide synthase
ns	Nonsignificant
ONE	4-oxo-2-nonenal
PC	Protein carbonylation
PMF	Primary myelofibrosis
PN	Protein nitrosylated
PUFA	Polyunsaturated fatty acyl
PV	Polycythemia vera
ROS	Reactive oxygen species
SOD	Superoxide dismutase
TBARS	Thiobarbituric reactive substances
Trx	Thioredoxin
VAD	Vincristine–Adriamycin–Dexamethasone
